# Perfusion practices and safety standards in Pakistan: Insights from a preliminary nationwide survey

**DOI:** 10.1051/ject/2025007

**Published:** 2025-06-16

**Authors:** Salman Pervaiz Butt, Nabeel Razzaq, Bill Cook, Babar Ali, Hashim Saqib, Aerfa Amir, Yazan Aljabery, Salman Abdulaziz, Arshad Ghori

**Affiliations:** 1 Manager Perfusion Services, Heart Vascular and Thoracic Institute, Cleveland Clinic Abu Dhabi PO BOX 112412 Abu Dhabi United Arab Emirates; 2 Clinical Perfusionist Heart Vascular and Thoracic Institute, Cleveland Clinic Abu Dhabi PO BOX 112412 Abu Dhabi United Arab Emirates; 3 Clinical Perfusionist, Perfusion Department, Glenfield Hospital Leicester LE3 9QP UK; 4 Department of Cardiac Perfusion Technology, Khyber Medical University 25100 Peshawar Pakistan; 5 Bashir Institute of Health Sciences, Shaheed Zulfiqar Ali Bhutto Medical University 44000 Islamabad Pakistan; 6 Rahbar Medical and Dental College Lahore 54810 Pakistan; 7 ECMO Task Force, Department of Health PO BOX 5674 20224 Abu Dhabi United Arab Emirates; 8 Anaesthesiology Institute, Cleveland Clinic Abu Dhabi PO BOX 112412 Abu Dhabi United Arab Emirates

**Keywords:** Perfusion safety, Cardiopulmonary bypass, Safety standards, Pakistan, Perfusion practices, Survey

## Abstract

*Introduction*: Perfusion safety in cardiothoracic surgery is critical, particularly in Pakistan where variability in practice standards exists. This survey investigates the current perfusion practices among Pakistani perfusionists, focusing on the adherence to safety standards during cardiopulmonary bypass (CPB) procedures. *Methods*: The survey was conducted over two weeks to explore key areas of perfusion practice, including the use of bubble detectors, level detectors, arterial filters, and saturation monitoring during CPB procedures. Out of approximately 350 practicing perfusionists in Pakistan, 66 responded, resulting in a response rate of 18.9%. The data was collected through an online platform, ensuring anonymity and voluntary participation. The survey included mainly Yes/No questions. To ensure reliability and validity, the questionnaire was reviewed by experts, pilot tested, and refined based on feedback, ensuring it was effective in gathering meaningful insights. *Results*: The survey results indicate a variable use of essential safety devices such as bubble and level detectors, arterial filters, and continuous venous saturation and cerebral saturation monitoring. While some perfusionists adhere to recommended safety protocols, gaps in the use of critical monitoring equipment were evident. *Conclusion*: The findings highlight the need for standardized perfusion practices in Pakistan to ensure safety and efficacy during CPB. Addressing the gaps in the use of safety and monitoring equipment could lead to improved patient outcomes. Further research is needed to explore the barriers to uniform safety standards and to develop strategies for enhancing perfusion safety across the country.

## Introduction

Cardiopulmonary bypass (CPB) is a critical component of cardiac surgeries, playing a crucial role in the outcome of such procedures. In Pakistan, where variations in medical practice standards are significant, ensuring the safety and effectiveness of CPB is particularly challenging. Perfusionists, who operate the heart-lung machines during these surgeries, have a pivotal role in managing these systems and their adherence to safety standards critically impacts patient outcomes. This survey is designed to assess current practices among Pakistani perfusionists regarding the use of monitoring and safety equipment during CPB procedures. It seeks to evaluate the extent to which essential safety and monitoring tools are employed, and to identify any discrepancies or commonalities in practice that could inform future improvements in the field. This initiative is part of a broader effort to standardize perfusion practices across the country to ensure high-quality and safe surgical outcomes.

## Methodology

This survey was conducted to explore several key areas of perfusion practice, including the routine use of bubble detectors, level detectors, arterial filters, continuous inline venous saturation monitoring, and cerebral saturation monitoring during CPB procedures. This sample size of this survey was primarily determined based on a two-week time frame. The survey collected data from 66 Perfusionists using an online platform targeting perfusionists across Pakistan.

Ethical considerations were upheld to ensure participant anonymity and voluntary participation. The survey consisted of multiple-choice and open-ended questions, allowing for the collection of both quantitative and qualitative data.

With an estimated total of 350 practicing perfusionists in Pakistan, the survey achieved a response rate of approximately 18.9%, representing a significant portion of the perfusion community. Conducted via an online platform, the survey targeted participants nationwide. While specific regional data was not collected due to the anonymous nature of the survey, efforts were made to encourage participation from diverse regions across the country.

To ensure the survey was reliable and valid, several important steps were taken before distribution. The questionnaire was reviewed by a panel of experts in perfusion and cardiopulmonary bypass practices to confirm that the questions accurately represented the intended topics and were relevant to the study’s goals. A pilot test was then conducted with a small group of perfusionists to check for clarity and relevance. Based on their feedback, the questionnaire was refined to improve its quality. Additionally, initial responses during data collection were monitored to ensure the survey remained aligned with the study’s objectives. These steps helped ensure that the survey was well-designed and capable of gathering reliable and meaningful data.

## Ethical considerations

In conducting this survey, ethical considerations were paramount. Anonymity and confidentiality were upheld, with no personally identifiable information collected or disclosed. Participation was voluntary, ensuring no coercion. Data collected was used solely for research purposes, maintaining privacy.

## Results

The Survey report “Perfusion Practices and Safety Standards in Pakistan: Insights from a Preliminary Nationwide Survey” was conducted over a period of two weeks, yielding a total of 66 responses.

The survey consisted of five structured questions focusing on adherence to safety standards, with most responses being (Yes/No) or conditional. While this format ensured clarity in data collection, it did not include demographic information such as age, gender, or experience, which could offer additional insights. Future studies should incorporate demographic variables to provide a more comprehensive understanding of factors influencing safety practices.

**Figure 1:**
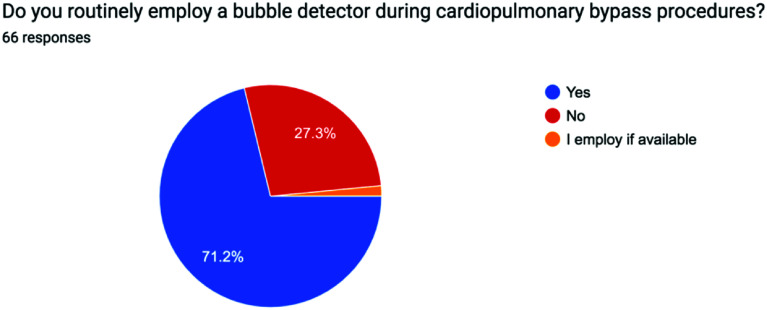
Pie chart showing use of Bubble alarm across Pakistan.

**Figure 2:**
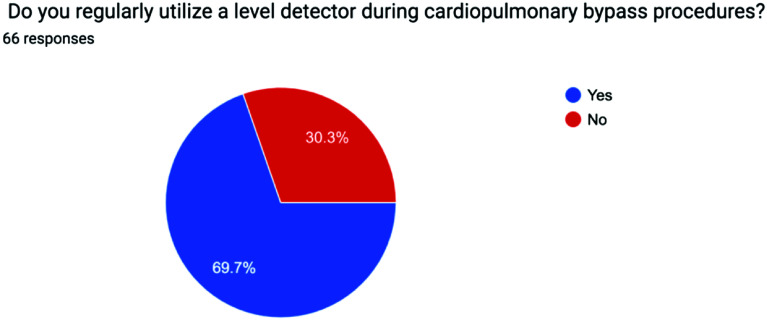
Pie chart showing use of Level alarm across Pakistan.

**Figure 3:**
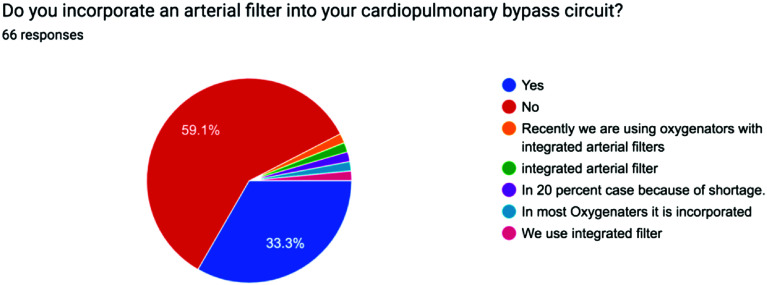
Pie chart showing use of Arterial filters across Pakistan.

**Figure 4:**
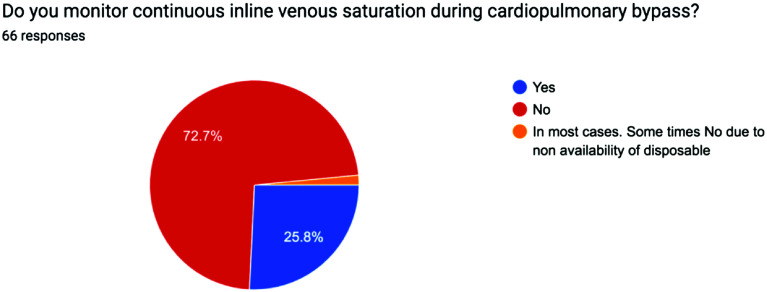
Pie chart showing use of Venous saturation monitoring across Pakistan.

**Figure 5:**
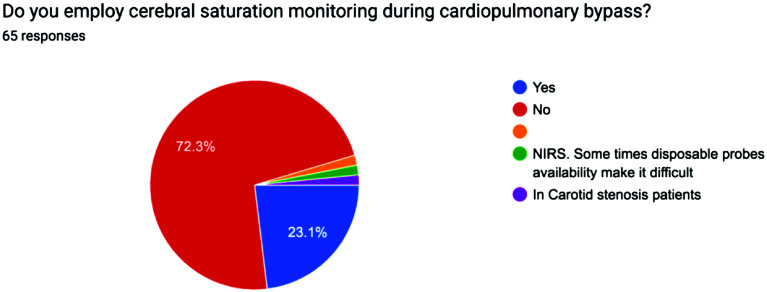
Pie chart showing use of Cerebral saturation across Pakistan.

The survey results were analyzed using descriptive statistics, with Yes/No responses reported as percentages and continuous variables expressed as mean values to ensure clarity and consistency. The distribution of responses for each question is visually represented in the accompanying pie charts, offering a clear snapshot of participants’ choices and opinions on the minimum standards of perfusion quality and safety in Pakistan from a professional perspective [1] (Figs. 1–5, Tables 1 and 2).

**Table 1:** Survey questions.

1	Do you routinely employ a bubble detector during cardiopulmonary bypass procedures?
2	Do you regularly utilize a level detector during cardiopulmonary bypass procedures?
3	Do you incorporate an arterial filter into your cardiopulmonary bypass circuit?
4	Do you monitor continuous inline venous saturation during cardiopulmonary bypass procedures?
5	Do you employ cerebral saturation monitoring during cardiopulmonary bypass?

**Table 2:** Survey results.

Question	Safety device	Yes	No	Conditional
1	Bubble detector	71.2%	27.3%	1.5% – only if available
2	Level detector	69.7%	30.3%	
3	Arterial filter	33.3%	59.1%	Some use integrated arterial filters
				Some depending on availability
4	Continuous venous saturation monitoring	25.8%	72.7%	Some have no availability of disposables
5	Cerebral saturation monitoring	23.1%	72.3%	Some use in selective cases of carotid stenosis
				Some when disposables were available

## Discussion

Safety features are paramount in providing good outcomes in CPB. They help prevent and manage problems that can occur during surgery. The heart lung machine is configured to sound an alarm and stop the arterial pump in the event of a high pressure, if there is too little volume in the venous reservoir, and when a bubble is detected. This prevents entry of an air embolus into the patient which can cause stroke and severe multiple organ dysfunctions. Saturation monitoring alarms are also often present in the heart lung machine are also seen as essential monitoring for the efficacy of the perfusion to the organs. Collectively these alarms can help improve perfusion of the patient, overall safety of the procedure, and ultimately better outcomes in the patient.

One of the main reasons a bubble alarm is used is to prevent gross air entrainment into the patient such as from emptying the reservoir, this is a catastrophic event where vast amounts of air is pumped into the arterial side of the patient, and can eventually lead to death. Gaseous microemboli (GME) may also arise from a number of sources during cardiac surgery such as manipulation of the aorta, cannulation, cross clamping, entrainment of air into the CPB venous line, during drug injection into the sampling manifold or from emptying the reservoir. Gaseous air is associated with covert cerebral insults, stroke and cognitive decline which has been seen in patients exposed with >500 GME than those exposed to less [1]. A survey by Kelting T surveyed 559 certified perfusionists looking at placement of the bubble alarm and their reasons [2]. Most people (35.6%) placed it on the outlet of the venous reservoir as this is felt to be the most likely source of gross air if the level alarm fails. 3.8% placed it between the arterial pump and oxygenator. 35.1% placed it between the oxygenator and arterial line filter and 23.6% distal to arterial filter. The reasoning being that both oxygenator and arterial filter are potential sources of where smaller bubbles may get caught and then passed on from. Specifically, placement distal to the arterial filter was seen as the protecting from all possible entry points of air. Nowadays integrated filters are common so a more recent study may show increased numbers of placement distal to the oxygenator as this will now be the most distal part of the circuit. The survey highlights many potential areas where a bubble alarm may be useful [3]. However, a study by Newlands et al comparing air bubble detectors from a Stockert S5 and S3 heart lung machine and an Emboli detection and classification quantifier found weak correlations between the two, suggesting the heart lung machine bubble detectors are not as clinically relevant when it comes to GME, but this does not negate its value for gross air detection. 27.3% of survey respondents of our survey stated they do not use bubble detectors in their unit. Although more recent studies may help our understanding in their use for GME, potentially perfusionists not using a bubble alarm may be exposing themselves to unrecognised emboli and certainly gross air [1].

Level detectors are essential for preventing air entrainment and maintaining proper fluid balance in the circuit. A catastrophic event may occur if the reservoir empties. The level alarm is the first alert the Perfusionist will receive in this event and can be seen as a major emergency if the issue goes unnoticed and air is pumped into the patient rendering initiation of a gross air protocol.

Also keeping a minimal level in the reservoir which is often recommended by each manufacturer helps prevent turbulent flow and GME from forming when running at low levels, a level alarm can help avoid and minimise these problems. 20 individuals stated they do not use level alarms in their centre. When looking at risk and reward in such a scenario, the risk to the patient can be potentially huge at the cost of a small alarm device.

Air micro-embolism reportedly occurs despite arterial filters of the cardiopulmonary bypass (CPB) circuit. Neurological complications occurring after open-heart surgery, which range from 1 to 6% for stroke and up to 76% for silent brain micro-infarcts or micro-bleeds. A relationship between air micro-embolism and cognitive decline affecting a variable percentage of patients after open-heart surgery has also been described. Gaseous microemboli may be one contributing factors to a multifactorial problem. Microemboli have been proven to be dangerous in experimental and clinical situations without any other confounding factors so awareness and prevention will certainly benefit patients. Putthetu et al did a study using an ultrasonic bubble counter (BCC300, Gampt) during bypass surgery in 10 CABG patients, and found that bubbles and air were detected throughout the bypass procedure. A vast majority of these are filtered through the oxygenator and number of bubbles decreased after the arterial filter. They found up to 2773 nl of total air going through their circuit in one procedure, up to 3424 bubbles detected in another procedure and bubbles larger than 1 mm detected. It was found that manipulations of the opened heart and arteries via direct blood-air contact, and manipulations on components of the CBP circuit are a major source of air in the CPB circuit [3].

Chung et al did a study where air volumes were measured in the middle cerebral artery territories using transcranial Doppler and found surgical manipulations, generated air bubbles in the brain [4], and Borger et al found a difference in neuropsychological outcome when comparing patients who underwent surgery with less than 10 perfusionist interventions versus those with 10 or more [5].

Although more evidence is needed to assess the clinical affects these microemboli have, evidence of microemboli created in surgery is still apparent. The growing use of low prime circuits to reduce haemodilution and systemic inflammatory response often now also see the use of vacuum assist devices which are also known to produce GME [6]. The use of Arterial filters can reduce systemic inflammatory responses or other neurological complications by filtering these embolis mentioned. While their use is relatively widespread, there exists variability, with some perfusionists using integrated filters within oxygenators [7]. 39 people from the survey said they do not use arterial filters and 1 said they do not use it 80% of the time. Thus, from a total of 66 people from the survey 60 people often do not use some sort of arterial filter whether incorporated or separate.

Central and mixed venous oxygen saturation monitoring has been known for guiding hemodynamic management in major surgeries. In paediatric cardiac surgery, perioperative goal-directed therapy with continuous ScVO_2_ monitoring is associated with excellent early survival and a low incidence of organ failure.

Low values of venous oxygen saturation during CPB are generally interpreted as an increased peripheral oxygen-extraction rate due to an oxygen delivery (DO_2_) inadequate to sustain the oxygen consumption (VO_2_). If DO_2_ falls below a critical value, settled at around 260 mL/min/m. a progressive increase of blood lactate is found, a marker of anaerobic energy production.

Rannuci et al did a study looking at 256 patients in paediatric cardiac surgery and their venous saturations and lactate levels. They found for ScVO_2_ values below 68%, with a significant increase of peak lactate during CPB predicted postoperative major morbidity. Patients who did not experience low values of ScVO_2_ and/or high values of peak lactate had an outcome free from adverse events in the great majority of the cases. This demonstrates the importance of measuring venous saturations during CPB. Only a quarter of survey respondents use venous saturation monitor in their centres. Venous Saturation monitoring measures the balance between oxygen delivery and demand of the patient. A statistically significant association between the S_v_O_2_ levels during CPB and 3-year survival after cardiac surgery has been seen demonstrating the potential benefits from such monitoring as it can flag up oxygen imbalances which may arise in surgery [8, 9].

Similarly, cerebral saturation monitoring is essential as it directly monitors the brains saturation. There was limited use of cerebral saturation monitoring found in this survey’s population 23.1% people. Monitoring is crucial for preventing neurological complications during CPB. It has been shown that maintaining an intact autoregulatory activity during CPB collectively with monitoring regional cerebral oxygen saturation will collectively contribute to optimization of patient care during CPB [10].

In recent years, near-infrared spectroscopy (NIRS) has been proposed as a surrogate of venous saturations in the setting of pediatric cardiac surgery. The main advantages are the continuous monitoring during and after the operation. The preoperative NIRS-derived regional oxygen saturation (rSO_2_) levels of <50% have been associated with an increased mortality in children undergoing congenital heart surgery. Demonstrating its usefulness [9].

The report from AmSECT’s International Consortium highlights essential minimum standards for perfusion practice. It recommends the routine use of safety devices like bubble detectors, level sensors, and arterial filters with audible and visual alarms to enhance patient safety. Continuous monitoring of key parameters, including arterial and venous pressures, SvO_2_, hematocrit, and blood gases, is crucial for maintaining perfusion quality. The report also emphasizes the importance of standardized institutional protocols, comprehensive procedural documentation, active participation in quality assurance programs, and regular maintenance of equipment to ensure reliability and safety in clinical practice [11].

According to the EACTS/EACTA/EBCP guidelines on cardiopulmonary bypass in adult cardiac surgery, it is strongly recommended to incorporate several key monitoring and safety features during CPB to enhance patient outcomes. A bubble detector should be used on all inflow lines to prevent air embolism, classified as Class I, Level C evidence. Continuous monitoring of mixed venous oxygen saturation (SvO_2_) and hematocrit (HCT) levels are essential to ensure adequate oxygen delivery and perfusion, supported by Class I, Level B evidence. The use of a level sensor is also recommended during procedures utilizing a (hard-shell) reservoir to monitor reservoir levels and prevent air from entering the circuit, classified as Class I, Level C evidence. Additionally, cerebral monitoring using near-infrared spectroscopy (NIRS) is advised, particularly in high-risk cases, to assess cerebral perfusion and oxygenation, classified as Class IIb, Level B evidence. Implementing these measures aligns with international guidelines and represents best practices for safety and quality in perfusion care [12].

A survey article by Anyasius Rutto et al. also highlights similarcritical challenges in Kenya and and provides recommendations for improving perfusion practices. It identifies a limited use of essential safety devices such as bubble detectors, level sensors, and arterial filters, raising concerns about patient safety during CPB procedures. The survey emphasizes a significant gap in formal training and certification for perfusionists, recommending the implementation of standardized education programs. It also underscores the absence of national guidelines and protocols, advocating for the development and adoption of comprehensive standards to ensure uniform safety practices. Resource constraints, including limited funding and availability of equipment, were noted as key barriers, with a call for increased collaboration between stakeholders and advocacy for resource allocation. These findings provide a roadmap for addressing safety, education, and resource challenges in perfusion practices, particularly in low-resource settings [13].

The barriers to consistent use of safety equipment, such as the availability of disposables and equipment, need to be addressed to ensure that all patients receive the highest standard of care. The survey results provide a nuanced view of the adherence to safety standards among perfusionists in Pakistan, revealing a landscape of mixed practices during cardiopulmonary bypass (CPB). Notably, the majority of respondents adhere to certain safety protocols, such as the routine use of bubble detectors and the monitoring of continuous venous saturation. These measures are critical for detecting air in the circuit and assessing the adequacy of oxygen delivery to the body, respectively, thus playing vital roles in ensuring patient safety during CPB. However, the survey highlights concerning gaps in the adoption of other safety technologies. A significant number of perfusionists reported not consistently using level detectors or cerebral saturation monitoring. while cerebral saturation monitors provide real-time data on brain oxygenation, serving as a crucial indicator of cerebral perfusion adequacy. The inconsistent use of these technologies suggests a potential risk area that could impact patient outcomes during Cardiac surgery in Pakistan.

These issues may also potentially be translated across to other countries with low income. Although cardiac surgery may be well established in certain places, budgeting issues and a lack of funding into perfusion departments would definitely have a knock-on effect into the level of safety a perfusionist can provide. Without thorough resource allocations the best output will never be achieved.

## Limitations

The survey has certain limitations that need to be acknowledged. The sample size, based on voluntary participation, may not fully represent the entire perfusion community, leading to potential selection bias. Additionally, the survey’s reliance on yes-and-no choices may limit the depth of responses and overlook important nuances. Furthermore, using individual perfusionists as the unit of analysis, rather than hospitals, could be seen as a limitation since practice is often influenced by a center’s protocols rather than individual preferences. The survey focused on five specific questions related to the use of safety devices in the circuit, which excluded broader aspects of standards, quality, and safety such as training, safety checklists, procedure-specific protocols, staffing, and duty hours. This narrow focus limits the scope of the insights obtained. To address these limitations, future research should consider more comprehensive sampling methods to ensure a representative sample of perfusion professionals. Incorporating open-ended questions and qualitative interviews can provide a deeper understanding of experiences and challenges. Moreover, exploring other safety features and monitoring specific outcomes in patients will provide a more holistic view. Future investigations should examine the specific factors contributing to any adverse outcomes in patients and the implications of different perfusion practices in diverse healthcare settings. By addressing these areas, future research can advance perfusion safety practices and enhance patient care in Pakistan.

## Conclusion

The survey highlights the need for standardization of perfusion practices in Pakistan to ensure the safety and quality of CPB procedures. Efforts should be made to provide perfusionists with the necessary resources and training to adhere to international standards. Further studies and interventions are required to address the gaps identified in this survey.

## Recommendations

1. **Training and Education:** Regular workshops and training sessions should be conducted to update perfusionists on the importance and use of monitoring equipment during CPB.
2. **Resource Allocation:** Hospitals and cardiac centers should ensure the availability of essential monitoring devices and disposables to all perfusion teams.
3. **Policy Development:** National guidelines should be developed to standardize perfusion practices across all cardiac centers in Pakistan.
4. **Outcome measuring:** Outcomes should be routinely measured to ensure the incorporation of safety devices are positively helping outcomes and to identify other areas of improvement.

## Data Availability

The research data are available on request from the authors.
